# A single-center experience of central nervous system tumors in children under three years old

**DOI:** 10.3389/fped.2024.1441016

**Published:** 2024-12-18

**Authors:** Junhua Wang, Chuanwei Wang, Zhimin Huang, Zhihua Zhang, Yuqi Zhang

**Affiliations:** ^1^ Department of Neurosurgery, Tsinghua University Yuquan Hospital (Tsinghua University Hospital of Integrated Traditional Chinese and Western Medicine), Beijing, China; ^2^ School of Clinical Medicine, Tsinghua University, Beijing, China; ^3^ Department of Neurosurgery, Qilu Hospital, Shandong University, Jinan, Shandong Province, China

**Keywords:** central nervous system, pediatrics, child, tumor, neurosurgery, chemotherapy

## Abstract

**Purpose:**

This study aims to summarize the characteristics of children under three years old (≤3 years) with central nervous system (CNS) tumors and to investigate the factors that influence their overall survival (OS) time.

**Methods:**

We treated 171 pediatric patients (≤3 years) with CNS tumors at Yuquan Hospital of Tsinghua University from January 2016 to June 2023. Of these, 162 cases were successfully followed up. Kaplan–Meier survival analysis and Cox regression were utilized to evaluate factors potentially influencing OS of malignancies.

**Results:**

There was a male predominance among the patients. The three most common tumors were embryonal tumors, gliomas, and craniopharyngiomas. Gross total resection (GTR) was achieved in select cases. Patients with high-grade malignancies were advised to undergo chemotherapy and/or radiotherapy after surgery. Optic gliomas and diffuse midline gliomas were partially resected and treated with adjuvant treatments. The median survival time of low-grade malignant tumors was 41.5 months, while that of high-grade malignant tumors was 15 months. Kaplan–Meier survival analysis identified the factors potentially influencing OS of malignancies: extent of resection, CNS WHO grade, grade of malignancies, and Ki-67 labeling index (Ki-67 LI). Subsequent multivariate analysis highlighted the interactive factor (extent of resection × CNS WHO grade) along with Ki-67 LI, as the most significant variables. Factors such as sex, age, tumor location, and onset-to-treatment time appeared not to affect OS.

**Conclusions:**

GTR remains the cornerstone of treatment for children (≤3 years) with CNS tumors, except for optic glioma, diffuse midline glioma, and germinoma. The interactive factor (extent of resection × CNS WHO grade) and Ki-67 LI are the most significant factors affecting OS. The implementation of preoperative neoadjuvant chemotherapy and early postoperative chemotherapy may enhance prognosis.

## 1 Introduction

Central nervous system (CNS) tumors were previously considered the second most common type of childhood tumors (1), but recent data from the United States now rank them as the most prevalent, surpassing leukemia (2). Approximately 8%–20% of CNS tumors in children occur in those under 3 years of age (1). The annual incidence rate of primary CNS tumors in children aged 0–3 years is 4.16 per 100,000 (3). The characteristics, treatments, and prognosis of CNS tumors in children ≤3 years differ from those in older children. We have compiled data on CNS tumors in children (≤3 years) treated at our hospital from 2016 to 2023 to provide more evidence for neurosurgeons and oncologists managing these patients.

## 2 Materials and methods

### 2.1 Clinical data

We treated 171 pediatric patients (≤3 years) with CNS tumors at Yuquan Hospital of Tsinghua University from January 2016 to June 2023. Of these, 168 underwent surgery, and 3 received chemotherapy without surgery. Inclusion criteria: (1) age (0–3 years); (2) sex (male and female); (3) CNS tumors treated in our hospital (no matter what therapies). Exclusion criteria: (1) patients having severe comorbidities preoperatively like organ dysfunctions, blood abnormalities, etc.; (2) patients or their guardians who did not have enough compliance with medical follow-up or medical research. The clinical manifestations, imaging features, operation conditions, pathological results and prognosis of these patients were analyzed retrospectively. The tumor classification was based on the 2016 WHO classification of tumors of CNS. All children's guardians had a detailed knowledge of the diagnosis, treatment, and prognosis, and signed the informed consent form. The study was approved by the ethnic committee of Yuquan Hospital of Tsinghua University (approval number: 2023029).

### 2.2 Research methods

Several factors were examined, including age, sex, histological tumor type, tumor location (supratentorial, subtentorial), clinical symptoms, (onset-to-treatment time), surgical outcomes [gross total resection (GTR), subtotal resection (STR), and partial resection (PR)], pathology (Ki-67 index, WHO grade, extent of malignancy), surgical complications, adjuvant treatments, and survival follow-up. Surgical strategies were tailored based on tumor location, size, malignancy, and growth characteristics. Ki-67 index (*x* < 5%, 5% ≤ *x* < 10%, 10% ≤ *x* < 30%, *x* ≥ 30%), and grade of malignancy [low-grade malignant tumors (LGMT, WHO Ⅰ-Ⅱ), high-grade malignant tumors (HGMT, WHO Ⅲ-Ⅳ)] were defined.

### 2.3 Multiple medical strategies

For benign tumors, we aimed for complete surgical resection. For high-grade malignancies, chemotherapy and operation were administered by doctors in our department, radiotherapy (RT) by external radiation oncologists, and three tactics of surgical resection of tumors were applied: gross total resection (GTR, complete resection), subtotal resection (STR, little residual nodule), and partial resection (PR, bigger residual mass) respectively based on MRI done less than 48 h postoperatively. For some of embryonal tumors, neoadjuvant chemotherapy (cisplatin, cyclophosphamide, and vincristine) was administered before surgery. After surgery, “vincristine + cyclophosphamide” and “carboplatin + etoposide” were used one after the other. Atypical teratoid rhabdoid tumors (AT/RT) were treated with “carboplatin + pirarubicin + cyclophosphamide” or “ifosfamide + carboplatin + etoposide”. Germinomas were typically treated with chemotherapy (carboplatin or cisplatin + etoposide) and RT. For teratomas and non-germinomatous germ cell tumors (NGGCTs), our approach was 1–2 cycles of chemotherapy (carboplatin or cisplatin + etoposide + ifosfamide), followed by surgery, additional chemotherapy, RT, and the remaining chemotherapy cycles. High-grade gliomas were treated within the maximum safe resection limits followed by postoperative chemotherapy (cisplatin + temozolomide) to prepare for RT. Low-grade gliomas underwent total resection when feasible, followed by chemotherapy (carboplatin + etoposide + vincristine) or follow-up based on pathology and extent of resection. Optic gliomas were typically partially resected, while total resection was attempted for focal endophytic brainstem gliomas where possible.

### 2.4 Follow-up

Survival data were collected through telephone and outpatient department surveys. The definition of overall survival (OS) is defined as the duration from treatment initiation to the last follow-up or death. The follow-up termination is defined as when the patient died, or censor occurred.

### 2.5 Statistical methods

Data analysis was performed using SPSS 20.0 (Chicago, USA). A *p*-value <0.05 was considered statistically significant. Kaplan–Meier survival analysis assessed the impact of individual factors on OS of malignancies, and multivariate Cox regression was used to evaluate interactive effects.

## 3 Results

### 3.1 Epidemiological characteristics

The ratio of males (103 cases) to females (68 cases) was 1.5:1. The age distribution included 22 infants (≤1 year, 13%), 67 toddlers (1 < *x* ≤ 2 years, 39%), and 82 young children (2 < *x* ≤ 3 years, 48%). The onset-to-treatment time ranged from 3 days to 2 years, with a median of 2 months.

### 3.2 Clinical manifestations

Clinical manifestations vary considerably owing to tumor location, size, and characteristics, hydrocephalus, brain edema, tumor hemorrhage, and other factors. Common symptoms include vomiting, unstable gait, headaches, limb weakness, nausea, and epilepsy, etc. Frequently observed signs are reduced limb muscle strength, gait disturbances, enlarged head circumference, positive pathological reflexes, impaired eye movements, posterior cranial nerve palsy, increased tension in the anterior fontanelle, visual acuity and field abnormalities, ataxia, abnormal postures, dysplasia, and developmental delays, etc. (Table 1).

**Table 1:** The symptoms and signs of the 171 patients.

Symtoms	*N.*	Percentage	Signs	*N.*	Percentage
Vomitting	43	25.1%	Reduced limb muscle strength	48	28.1%
Unstable walking	35	20.5%	Gait disturbances	19	11.1%
Headache	21	12.3%	Enlarged head circumference	15	8.8%
Limb weakness	18	10.5%	Positive pathological reflexes	15	8.8%
Nausea	17	9.9%	Dlipopia	12	7.0%
Epilepsy	13	7.6%	Posterior cranial nerve palsy	11	6.4%
Strabismus	9	5.3%	Increased tension in the anterior fontanelle	10	5.8%
Big head	7	4.1%	Visual acuity and field abnormalities	10	5.8%
Torticollis	6	3.5%	Ataxia	8	4.7%
Accidentally discovery	6	3.5%	Abnormal postures	7	4.1%
Blurred vision	5	2.9%	Dysplasia	6	3.5%
Somnolence	4	2.3%	Nystagmus	5	2.9%
Giggle	4	2.3%	Sunset sign	5	2.9%
Bucking	3	1.8%	Pupillary abnormality	5	2.9%
Subcutaneous mass	3	1.8%	Neck rigidity	4	2.3%
Polydispsia and Polyuria	2	1.2%	Facial paralysis	4	2.3%
Alalia	1	0.6%	Malnutrition	3	1.8%
			Unconsciousness	3	1.8%

### 3.3 Surgical and adjuvant treatments

Patients were categorized based on the extent of surgical resection into three groups: GTR (116 cases), STR (32 cases), and PR (20 cases). For those with malignant tumors after surgery, treatment choices varied; some guardians opted for chemotherapy alone, while others selected a combination of RT and chemotherapy. Three patients with germinomas received both chemotherapy and RT without undergoing surgery. Following standard adjuvant therapy, tumors in three patients disappeared and showed no signs of recurrence at follow-up.

### 3.4 Pathological results

**Figure 1:**
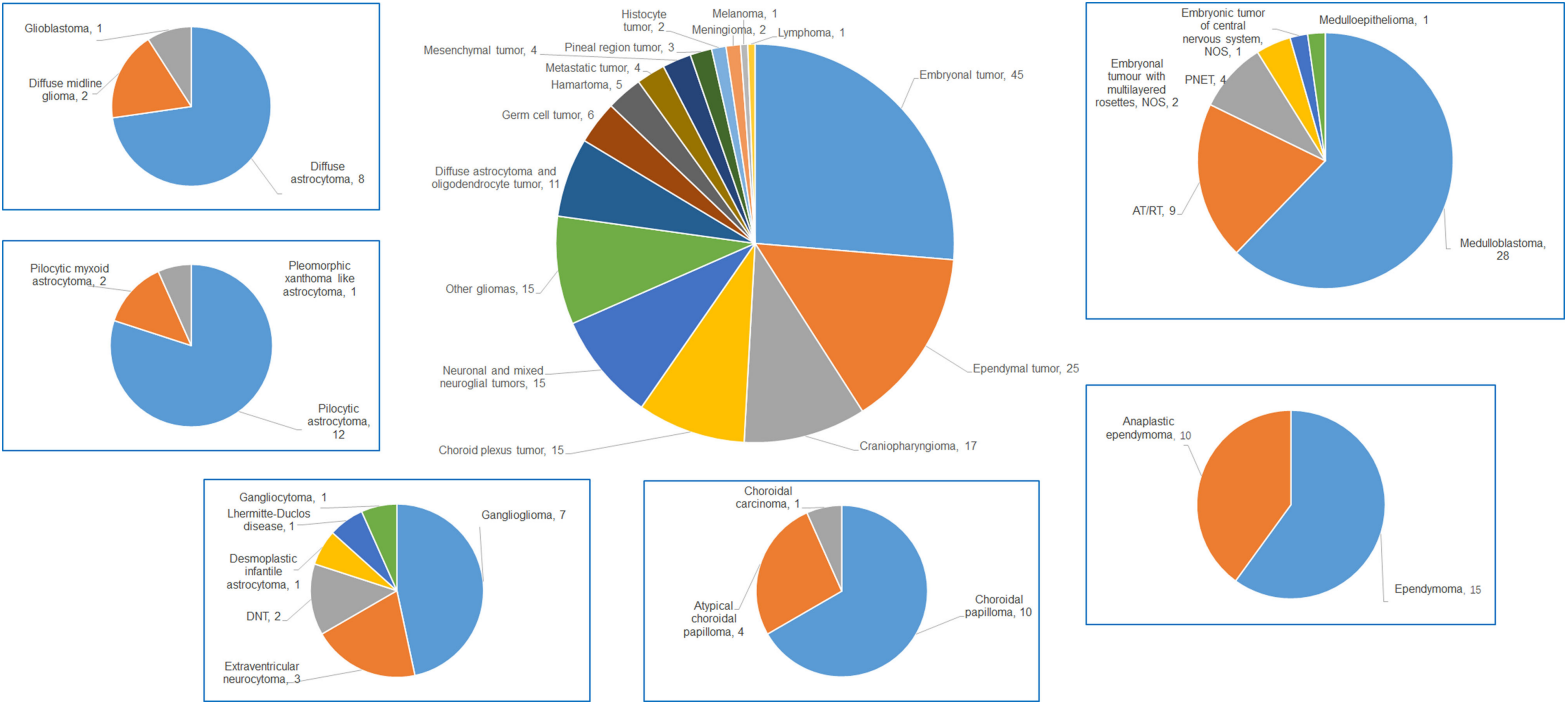
The pie chart of all types of children (≤3 years) with CNS tumors and details of the top 6 malignancies in our data.

The study encompassed a broad spectrum of pathological types, including 16 categories and 38 subtypes. The seven most prevalent categories were embryonal tumors (45 cases), ependymal tumors (25 cases), craniopharyngiomas (17 cases), choroid plexus tumors (15 cases), other astrocytomas (15 cases), neuronal and mixed neuroglial tumors (15 cases), and diffuse astrocytic and oligodendrocytic tumors (11 cases). The specifics are detailed in Table 2 and Figure 1.

**Table 2:** The spectrum of CNS tumor in child (≤3 years) and their locations.

	The fourth ventricle and cerebellar vermis	Cerebellar hemisphere	Cerebello-pontine angle	Brain stem	The lateral ventricle	The third ventricle	Cerebral hemisphere	Sellar	Basal ganglia	Pineal Region	Skull base	Spinal	Total
**Embryonal tumor**	**28**	**4**	**0**	**2**	**1**	**0**	**7**	**0**	**1**	**1**	**0**	**1**	**45**
Medulloblastoma	25	3	0	0	0	0	0	0	0	0	0	0	28
AT/RT	3	1	0	0	0	0	4	0	1	0	0	0	9
PNET	0	0	0	1	1	0	1	0	0	0	0	1	4
Embryonal tumour with multilayered rosettes, NOS	0	0	0	1	0	0	1	0	0	0	0	0	2
Embryonic tumor of central nervous system, NOS	0	0	0	0	0	0	0	0	0	1	0	0	1
Medulloepithelioma	0	0	0	0	0	0	1	0	0	0	0	0	1
**Ependymal tumor**	**14**	**0**	**2**	**1**	**1**	**0**	**6**	**0**	**0**	**0**	**0**	**1**	**25**
Ependymoma	3	0	1	0	1	0	4	0	0	0	0	1	10
Anaplastic ependymoma	11	0	1	1	0	0	2	0	0	0	0	0	15
**Sellar tumor**	**0**	**0**	**0**	**0**	**0**	**2**	**0**	**15**	**0**	**0**	**0**	**0**	**17**
craniopharyngioma	0	0	0	0	0	2	0	15	0	0	0	0	17
**Choroid plexus tumor**	**3**	**0**	**0**	**0**	**12**	**0**	**0**	**0**	**0**	**0**	**0**	**0**	**15**
Choroidal papilloma	1	0	0	0	9	0	0	0	0	0	0	0	10
Atypical choroidal papilloma	0	0	0	0	1	0	0	0	0	0	0	0	1
Choroidal carcinoma	2	0	0	0	2	0	0	0	0	0	0	0	4
**Neuronal and mixed neuroglial tumors**	**0**	**4**	**0**	**0**	**0**	**0**	**8**	**2**	**1**	**0**	**0**	**0**	**15**
Ganglioglioma	0	2	0	0	0	0	5	0	0	0	0	0	7
Extraventricular neurocytoma	0	0	0	0	0	0	2	1	0	0	0	0	3
DNT	0	0	0	0	0	0	1	0	1	0	0	0	2
Desmoplastic infantile astrocytoma	0	0	0	0	0	0	0	1	0	0	0	0	1
Lhermitte-Duclos disease	0	1	0	0	0	0	0	0	0	0	0	0	1
Gangliocytoma	0	1	0	0	0	0	0	0	0	0	0	0	1
**Other gliomas**	**2**	**2**	**0**	**1**	**0**	**1**	**1**	**8**	**0**	**0**	**0**	**0**	**15**
Pilocytic astrocytoma	2	2	0	1	0	1	0	6	0	0	0	0	12
Pilocytic myxoid astrocytoma	0	0	0	0	0	0	0	2	0	0	0	0	2
Pleomorphic xanthoma like astrocytoma	0	0	0	0	0	0	1	0	0	0	0	0	1
**Diffuse astrocytoma and oligodendrocyte tumor**	**0**	**3**	**0**	**2**	**0**	**1**	**3**	**0**	**2**	**0**	**0**	**0**	**11**
Diffuse astrocytoma	0	3	0	0	0	1	2	0	2	0	0	0	8
Diffuse midline glioma	0	0	0	2	0	0	0	0	0	0	0	0	2
Glioblastoma	0	0	0	0	0	0	1	0	0	0	0	0	1
**Hamartoma**	**0**	**0**	**0**	**0**	**0**	**0**	**0**	**5**	**0**	**0**	**0**	**0**	**5**
Hamartoma of hypothalamus	0	0	0	0	0	0	0	5	0	0	0	0	5
**Germ cell tumor**	**0**	**0**	**0**	**0**	**0**	**2**	**0**	**1**	**1**	**2**	**0**	**0**	**6**
Germinoma	0	0	0	0	0	0	0	0	1	1	0	0	2
Mixed germ cell tumor	0	0	0	0	0	0	0	1	0	1	0	0	2
Malignant teratoma	0	0	0	0	0	2	0	0	0	0	0	0	2
**Metastatic tumor**	**0**	**0**	**0**	**0**	**1**	**0**	**3**	**0**	**0**	**0**	**0**	**0**	**4**
Brain metastasis of hepatoblastoma	0	0	0	0	1	0	3	0	0	0	0	0	4
**Mesenchymal tumor**	**0**	**0**	**0**	**1**	**0**	**0**	**1**	**0**	**0**	**0**	**1**	**1**	**4**
Hemangioma	0	0	0	1	0	0	1	0	0	0	1	0	3
Lipoma	0	0	0	0	0	0	0	0	0	0	0	1	1
**Pineal region tumor**	**0**	**0**	**0**	**0**	**0**	**0**	**0**	**0**	**0**	**3**	**0**	**0**	**3**
Pinealoblastoma	0	0	0	0	0	0	0	0	0	3	0	0	3
**Histocyte tumor**	**0**	**0**	**0**	**0**	**0**	**0**	**2**	**0**	**0**	**0**	**0**	**0**	**2**
Langerhans cell histiocytosis	0	0	0	0	0	0	2	0	0	0	0	0	2
**Meningioma**	**0**	**0**	**0**	**0**	**0**	**0**	**0**	**0**	**0**	**0**	**2**	**0**	**2**
Invasive meningioma	0	0	0	0	0	0	0	0	0	0	2	0	2
**Melanoma**	**0**	**0**	**0**	**0**	**0**	**0**	**1**	**0**	**0**	**0**	**0**	**0**	**1**
Melanoma	0	0	0	0	0	0	1	0	0	0	0	0	1
**Lymphoma**	**0**	**0**	**0**	**0**	**0**	**0**	**0**	**0**	**1**	**0**	**0**	**0**	**1**
Diffuse large B-cell lymphoma	0	0	0	0	0	0	0	0	1	0	0	0	1
**Total**	47	13	2	7	15	6	32	31	6	6	3	3	**171**

The bold values mean the 16 categories which are differentiated from the 38 subtypes.

### 3.5 Follow-up results and statistical results

**Figure 2:**
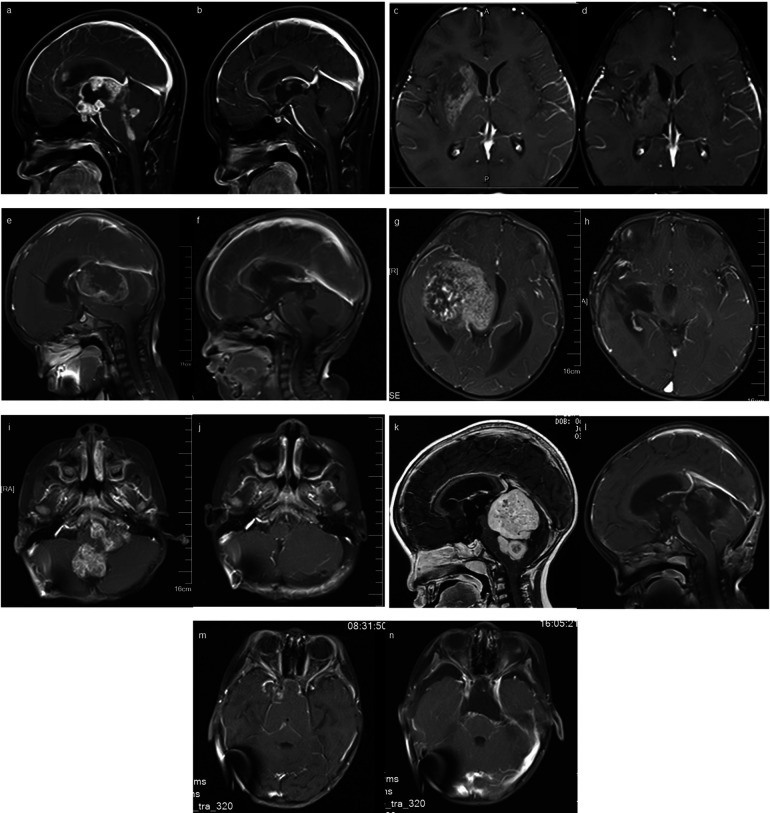
The typical preoperative and follow-up MRI images of several patients. a and b: CNS germinoma metastasis, before and after “chemotherapy and RT”; c and d: CNS germinoma in basal ganglia, before and after “chemotherapy and RT”; e and f: pinealoblastoma, before and after “operation + chemotherapy and RT”; g and h: high-grade malignant astrocytoma, before and after “operation + chemotherapy and RT”; i and j: ependymoma, before and after “operation + RT”; k and l: medulloblastoma, before and after “operation + chemotherapy and RT”; m and n: craniopharygioma, before and after “operation”;.

Out of 171 patients, nine were lost to follow-up. The remaining 162 patients were successfully monitored. Survival times ranged from 2 to 87 months, with a median OS time of 31.5 months. Follow-up duration ranged from 1 to 90 months. Follow-up interval is once every three months for malignancies and half a year for benign tumors. Some typical follow-up MRI images were presented in Figure 2. The recurrence rate of benign tumors was 23.1% (6/26). The median survival time of malignancies was 23.5 months, while that of low-grade malignant tumors was 41.5 months and high-grade malignant tumors 15 months. During the follow-up period, 43 patients passed away. The survival data of malignancies were analyzed, including LGMT (WHO Ⅰ–Ⅱ, 60 cases) and HGMT (WHO Ⅲ–Ⅳ, 76 cases). We excluded benign tumors (craniopharyngiomas, hamartoma of hypothalamus, langerhans cell histiocytosis, lipoma, hemangioma) out of the statistical analysis. Kaplan–Meier survival analysis identified several key factors potentially impacting patient OS: extent of resection, CNS World Health Organization (WHO) grade, grade of malignancies (LGMT and HGMT), and Ki-67 labeling index (Ki-67 LI) (Table 3). Considering there may be an interactive impact between extent of resection and CNS WHO grade, the “extent of resection × CNS WHO grade” was also be introduced into Cox regression analysis. Subsequent multivariate analysis highlighted the interactive factor (extent of resection × CNS WHO grade) and Ki-67 LI, as the most critical variables influencing survival (Table 4). Factors such as sex, age, location, and onset-to-treatment time were not statistically significant.

**Table 3:** The univariate analysis by Kaplan–Meier plots.

Variable	Number	Event	*P*
Age (years)			=0.932
x ≤ 1	18	4	
1 < x ≤ 2	56	18	
2 < x ≤ 3	62	19	
Sex			=0.921
male	81	26	
female	55	15	
Onset-to-treatment time (months)			=0.381
x < 1	27	8	
1 ≤ x < 3	54	17	
3 ≤ x < 6	23	6	
6 ≤ x < 12	19	8	
x ≥ 12	13	2	
Location of tumor			=0.144
supratentorial	71	17	
subtentorial	65	24	
Extent of Resection			=0.002
total resection	92	20	
subtotal resection	29	11	
partial resection	15	10	
WHO classification			<0.001
Ⅰ	31	2	
Ⅱ	29	1	
Ⅲ	27	10	
Ⅳ	49	28	
Extent of malignancy			<0.001
low-grade	60	3	
high-grade	76	38	
Ki-67 index			<0.001
*x* < 5	14	1	
5 ≤ *x* < 10	45	2	
10 ≤ *x* < 30	36	11	
*x* ≥ 30	41	27	

**Table 4:** The multivariate analysis by Cox regression.

Factor	Sig.	Exp (B)	95.0% CI	
			Down	Up
Ki-67 LI	0.000	3.06	1.831	5.130
Resection*WHO	0.001	1.20	1.073	1.341

## 4 Discussion

The epidemiology, treatments, and prognosis of CNS tumors in children ≤3 years differ significantly from those in older children and adults. In this age group, CNS tumors frequently occur along the cranial midline. Supratentorial cases (*n* = 104) were more common than infratentorial ones (*n* = 67), with a ratio of 1.55:1. Details on tumor types and locations are provided in Table 2. The three most prevalent CNS tumors in children under 15 years are low-grade gliomas (36.4%), high-grade gliomas (22.3%), and embryonal tumors (18.7%) (4). Studies indicate that in children ≤3 years, astrocytomas are most common, followed by medulloblastoma (MB) (5, 6). However, a study of 86 pediatric patients in this age group showed that embryonal tumors were most prevalent (37.2%), followed by astrocytomas (31.4%) (7). Data from 81 cases revealed no gender predominance, and the top six most common diseases were astrocytoma (21.0%), MB (19.8%), ependymoma (EM, 16.1%), choroid plexus papilloma (CPP, 8.6%), primitive neuroectodermal tumor (PNET, 8.6%), and atypical teratoid/rhabdoid tumor (AT/RT, 7.4%) (3). Overall, embryonal tumors are the most frequently occurring CNS tumor in children (≤3 years) and in children (≤4 years) (2, 3). Our data showed that embryonal tumors, with 45 cases (26.3%), were the most common, followed by all types of astrocytomas with 26 cases (15.2%). MB with 28 cases (16.4%) was the first subtype. Variations in incidence rates across different studies may be attributed to biases related to patient sources in various countries and regions.

The three most common symptoms observed in this study were vomiting, unstable walking, and headache. These may be attributed to several characteristics of infant brain tumors, which are typically large, centrally located along the midline, and frequently associated with hydrocephalus. Symptoms in children (≤3 years) may be atypical; however, physical examinations often reveal significant findings, including decreased limb muscle strength (48 cases), increased head circumference (18 cases), and abnormal vision (18 cases). These findings correspond to related symptoms such as limb weakness and reduced activity (18 cases), enlarged head (7 cases), and diminished vision (5 cases). This phenomenon may be linked to the challenges infants and toddlers face when expressing discomfort, the possibility that some parents may not closely monitor their children, and the fact that the initial healthcare providers these children see may not be specialized pediatric neurologists. These factors can lead to delayed diagnosis and treatment. Early diagnosis of CNS tumors in children (≤3 years) is crucial for effective treatment.

**Figure 3:**
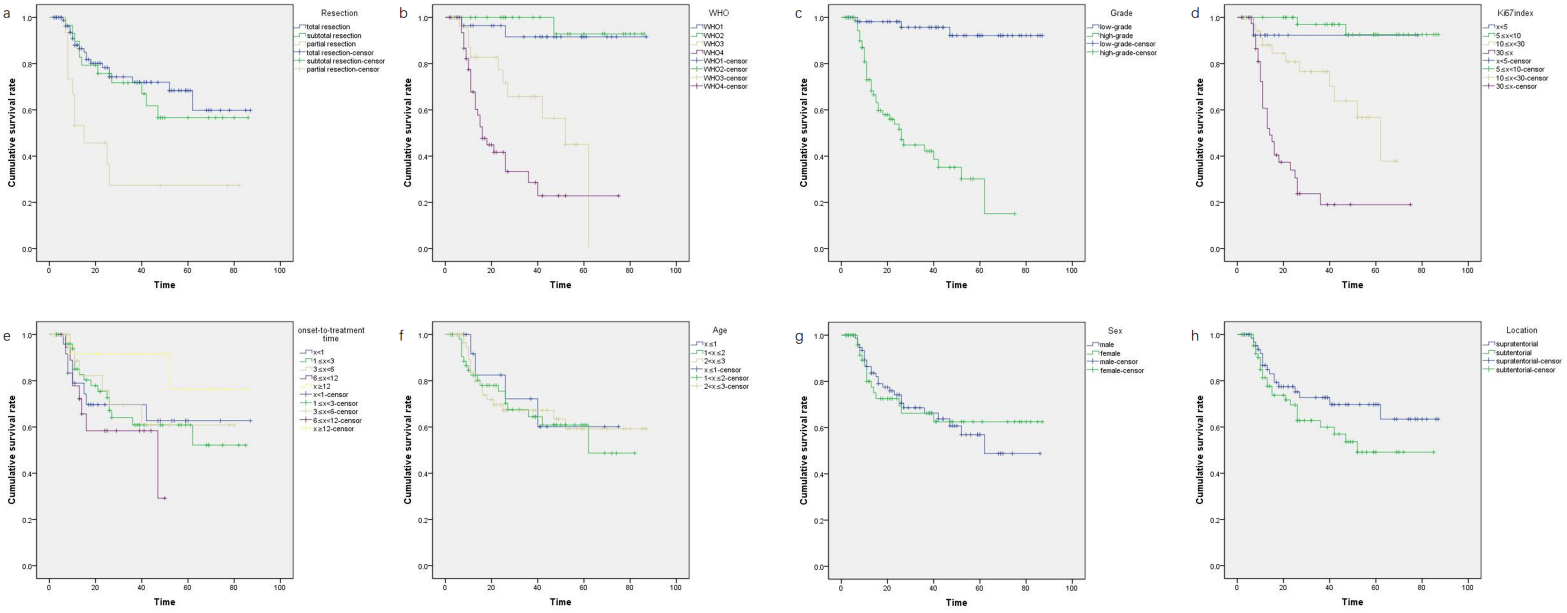
The Kaplan–Meier plots on four significant factors affecting survival time of children (≤3 years) with CNS tumors: figure a for extent of resection: gross total resection, subtotal resection, partial resection, *p* = 0.002; figure b for CNS WHO grade: WHO Ⅰ, WHO Ⅱ, WHO Ⅲ, WHO Ⅳ, *p* < 0.0001; figure c for grade of malignancies: low-grade, high-grade, *p* < 0.0001; figure d for Ki-67 LI: *x* < 5, 5 ≤ *x* < 10, 10 ≤ *x* < 30, *x* ≥ 30, *p* < 0.0001. The Kaplan–Meier plots on four not significant factors affecting survival time of children (≤3 years) with CNS tumors: figure e for age (year): *x* ≤ 1, 2 < *x* ≤ 3, 1 < *x* ≤ 2, 2 < *x* ≤ 3, *p* = 0.932; figure f for sex: male, female, *p* = 0.921; figure g for onset-to-treatment time (month): *x* < 1, 1 ≤ *x* < 3, 3 ≤ *x* < 6, 6 ≤ *x* < 12, *x* ≥ 12, *p* = 0.381; figure h for location: supratentorial; subtentorial; *p* = 0.144.

All the benign tumors such as craniopharyngioma survived at the end of follow-up period and were excluded from survival analysis. The OS of benign tumors are almost determined by surgery. Considering the survival time of patients with malignancies (136 cases), several potential factors were analyzed, including age, sex, onset-to-treatment time, tumor location, extent of resection, CNS WHO grade, grade of malignancies, and Ki-67 LI. Statistical tests were applied to all these factors, except for RT and chemotherapy, due to insufficient data for a meaningful statistical analysis. The results indicated no significant differences in prognosis based on age (*x* ≤ 1, 1 < *x* ≤ 2, and 2 < *x* ≤ 3 years, *p* = 0.932), sex (male, female, *p* = 0.921), location of tumors (supratentorial, subtentorial, *p* = 0.144), and onset-to-treatment time (*x* < 1, 1 ≤ *x* < 3, 3 ≤ *x* < 6, 6 ≤ *x* < 12, *x* ≥ 12 months, *p* = 0.381). While some researchers believe that younger children (≤3 years) with CNS tumors have poorer prognoses than older children and adults, others find no differences (1, 3–5, 8–10). Regardless of prognosis or recurrence rates, children (≤3 years) may face more neuropsychological and cognitive challenges than older children (1, 3, 11–13). A clinical study indicated that prognostic factors for patients (≤3 years) with CNS malignancies include tumor location and histopathology, showing a better OS rate of 40.9% for tumors in the posterior cranial fossa than that of 68.1% for supratentorial locations after appropriate treatments (7). Our data show different trends, with a higher OS rate of 76.5% for patients with supratentorial malignancies compared to 63.9% for those with subtentorial malignancies, which showed no significant difference (*p* = 0.144, Figure 3).

Our data showed that the CNS WHO grade of the tumor (*p* < 0.0001, Figure 3) and Ki-67 LI (*p* < 0.0001, Figure 3) may affect the survival time of children (≤3 years) with CNS tumors. The OS for children with highly malignant CNS tumors (WHO Ⅲ: 42.6 ± 4.86; WHO Ⅳ: 29.9 ± 4.38) was significantly lower than that for children with low-grade malignant tumors (WHO Ⅱ: 83.2 ± 2.68; WHO Ⅰ:83.5 ± 2.39, *p* < 0.0001, Figure 3). This indicated that highly malignant CNS tumors in children (≤3 years) significantly impact survival time, whereas the prognosis for low-grade malignancies is more favorable. This finding aligns with previous literature (14). Regarding Ki-67 LI, we categorized it into four groups (*x* < 5, 5 ≤ *x* < 10, 10 ≤ *x* < 30, *x* ≥ 30). OS was higher in groups 1 and 2 compared to group 3, and group 3 showed better OS than groups 4, suggesting that Ki-67 LI levels of 10 and 30 may represent critical thresholds (*p* < 0.0001, Figure 3). Gliomas can manifest in various forms in children (≤3 years). Glioblastoma (GBM), rare in this age group, was recorded in only one case in our series. The U.S. Surveillance, Epidemiology, and End Results (SEER) program noted that in children under four years, factors like supratentorial location, gross total resection (GTR), and more recent year of diagnosis are associated with improved survival rates, whereas sex, race, region, or tumor size showed no significant correlation with primary outcomes (15). Another study suggested that children (≤5 years) with GBM tend to have longer survival times than older children (>5 years) (16). Conversely, the 5-year survival rate for children (≤3 years) with low-grade malignant gliomas is reportedly similar to that in older children (17, 18). The survival rate for gliomas in children (≤3 years) varies widely from 20% to 90%, with an average OS rate of 46%; high-grade astrocytomas are more prevalent than low-grade astrocytomas in this age group (4, 13, 15–17, 19). However, in our series, low-grade astrocytomas (17 cases) predominated over high-grade astrocytomas (11 cases). What we should know is that some young children with low-grade gliomas can get a good outcome by RT, chemotherapy or targeted therapy (20). So, we should not pursue GTR in some special patients suffering optic glioma (mainly consisting of low-grade gliomas), to protect their visual function. In fact, efforts trying to achieve total resection may destroy the vision in the patients with optic glioma.

According to univariate analysis, patients with malignancies who underwent GTR generally had good OS (*p* < 0.002, Figure 3). Previous studies suggested that for children (≤3 years) with malignant CNS tumors, if total surgical resection is achieved and there is a favorable response to chemotherapy, RT can be postponed until at least age 4 without affecting survival times (21). However, simply considering clinically, we thought that even with GTR, pediatric patients with high-grade malignancies such as group 3 MB, AT/RT, PNET and metastasized malignancies may still have a poor prognosis, a finding also observed in previous studies (3, 10). These types are also some of the predominated malignant types in infants and toddlers (10). From another viewpoint, a longer OS does not mean a better quality of life or cognitive function, and we cannot get data about medical comorbidities and quality of life even in the SEER database (10, 15). We introduced three interactive factors (extent of resection × CNS WHO grade, extent of resection × Ki-67 LI, extent of resection × grade of malignancies) and four single factors (extent of resection, CNS WHO grade, grade of malignancies, Ki-67 LI) into the Cox regression analysis. Statistically, two main factors significantly impacted OS (*p* = 0.001, Figure 3): the interactive factor (extent of resection × CNS WHO grade) and Ki-67 LI. This indicates that single factor “GTR” may not be so certainly beneficial to the prognosis of patients who suffer high-grade malignancies. There is a strong mutual interaction between GTR and CNS WHO grade on influencing prognosis of patients, because highly malignant nature is against the effect of total resection. Aggressive GTR may not always be necessary in young children with high malignancy rates especially when the tumor locates in the key area such as basal ganglia, thalamus or brain stem. Alternative treatments such as RT, chemotherapy, targeted therapy, immunotherapy, and other agents may be beneficial (5, 13, 17, 20, 22–30). Furthermore, total excision should be avoided for certain malignant tumors like optic glioma, diffuse midline glioma, and germinoma. Typically, optic glioma requires PR to relieve pressure and protect vision, followed by RT, chemotherapy, or targeted therapy (20, 31). Germinoma is highly responsive to RT and chemotherapy and often does not require surgical intervention (24, 29). Patients with diffuse midline glioma may benefit from next-generation sequencing (NGS) and targeted drugs instead of aggressive resection (1, 8, 9, 13–15, 17, 19, 26).

Recently, adjuvant therapies such as chemotherapy have become increasingly important in treating children (≤3 years) with malignant CNS tumors. Clinical trials like Head Start I, II, and III have demonstrated that the following post-surgery regimens can improve prognosis and minimize the side effects of RT for children (≤3 years) with MB, regardless of whether the tumors have metastasized at initial diagnosis: (1) induction chemotherapy + intensive myeloablative chemotherapy + autologous hematopoietic progenitor cell salvage treatment; (2) intraventricular methotrexate injection (IT-MTX) + cerebral and spinal cord irradiation (CSI) (23, 32). High-dose chemotherapy combined with autologous stem cell rescue therapy has significantly improved survival rates in children (≤3 years) with high-risk or recurrent MB and supratentorial PNET, thus delaying or avoiding RT (27, 33, 34). In our experience with specific chemotherapy strategies for young children (≤3 years), we utilized intraventricular methotrexate injections combined with conventional chemotherapy in two MB patients after surgery. These two patients did not receive RT until the age of 3 years, and no recurrences were observed during follow-up. We also administered preoperative neoadjuvant chemotherapy in three MB and two NGGCT cases, which reduced tumor blood supply and size, facilitating GTR. Furthermore, we employed chemotherapy early post-surgery in children (≤3 years) with malignant CNS tumors like MB, AT/RT, and NGGCT. Although not statistically verified, we believe this approach may improve survival times, which is consistent with the literature (22). Long-term follow-ups and additional cases are needed to consolidate these findings.

Many scholars believe that RT is not recommended for young children (especially those ≤3 years) with CNS malignancies, as it may cause intellectual and cognitive impairment, neuropsychological sequelae, and growth retardation. Consequently, chemotherapy has gained prominence as an adjuvant treatment (21, 35, 36). Our experience with RT for children (≤3 years) is limited. However, recent literature offers some hope. A large-scale study involving 2,996 young patients (≤2 years) with CNS tumors found that, except for choroid plexus tumors, the survival time for children with other malignant CNS tumors significantly improved after RT (10). Yet, extended survival does not necessarily enhance quality of life, and the benefits of RT must be balanced against its side effects (10). A comprehensive, prospective, multisite, longitudinal clinical trial in North America and Australia examined 139 infants with CNS malignancies and determined that changes in cognitive function depended more on tumor location and surgical techniques than on RT (12). For ependymomas, proton RT has been deemed safe and effective for children under 3 years (37). Additionally, a study reported that intensity-modulated RT (IMRT) for children with anaplastic ependymoma (≤3 years) achieved better local control (30). This evidence suggests that high-precision RT using modern techniques can be effectively applied as adjuvant therapy for young children (≤3 years) with malignant CNS tumors. For children (≤3 years) with CNS malignancies (including MB, PNET, and ependymoma), a comprehensive treatment plan combining RT, induction chemotherapy, consolidated high-dose myeloablative chemotherapy (HDC), and autologous stem cell rescue therapy (AuSCR) is employed, achieving a 5-year OS rate of 60%–70% (38–40).

With the advent of the molecular era, gene detection has significantly advanced the standardized diagnosis, prognostic evaluation, and treatment of various CNS malignancies. Taking MB as an example, the Sonic Hedgehog (Shh) type predominates in infant MB cases, while Wnt types are less common, and group 3 and group 4 types are exceedingly rare (25, 41). A multi-center clinical phase II trial reported that infants with Shh-*γ* MB benefit from systemic chemotherapy following surgery, and most children with the Shh subtype of MB can be effectively managed with conservative RT (42). The HIT-2000 clinical trial reported that systemic chemotherapy combined with intraventricular injection of methotrexate is a viable adjuvant therapy for infants with non-metastatic Shh-β MB after surgery, achieving a 93% 5-year PFS and 100% OS (43). It has also been noted that group 3 and group 4 infant MB can be treated with systemic chemotherapy + low-dose CSI or systemic chemotherapy + local irradiation; however, the prognosis for these subtypes remains generally poor, regardless of the treatment regimen. Innovative approaches, such as low-dose proton beam CSI, targeted therapy using radioactive element-binding monoclonal antibodies, or chimeric antigen receptor T-cell therapy, are beginning to enter clinical trials, though their efficacy is yet to be confirmed in large-scale studies (28, 42, 44). A retrospective analysis of CNS malignancies in children under 3 years old demonstrated that a sequential treatment regime of induction chemotherapy, local RT, and maintenance chemotherapy can yield a 5-year PFS of 100% for Shh *γ* MB, and 50% for other types of MB and other malignant CNS tumors (45). Based on our experience and the literature, appropriate chemotherapy indeed benefits malignancies in children (≤3 years).

This study has several limitations. First, the CNS WHO grade and grading of CNS tumors were not based on the latest criteria published in 2021, because many cases were treated before 2021 in this study. Second, the effects of RT and chemotherapy on OS were not statistically assessed due to insufficient data. Third, this was a single-center study and not a comprehensive multicenter investigation. Despite these limitations, we believe that our findings can still offer valuable insights to others.

## 5 Conclusion

The three most common CNS tumors in children under 3 years old in our study were embryonal tumors, gliomas, and craniopharyngiomas. GTR remains the primary treatment option, except for optic gliomas, diffuse midline gliomas, and germinomas. Univariate analysis identified the factors that influenced OS: extent of resection, CNS WHO grade, grade of malignancies, and Ki-67 LI. Further multivariate analysis highlighted the interactive factor (extent of resection × CNS WHO grade) and Ki-67 LI as the most critical variables. Children (≤3 years) with high-grade malignancies continue to have a poor prognosis, whereas those with low-grade malignancies and benign tumors typically experience significantly longer lifespans. The use of preoperative neoadjuvant chemotherapy and early postoperative chemotherapy may improve the prognosis of children (≤3 years) with malignant CNS tumors.

## Data Availability

The original contributions presented in the study are included in the article/Supplementary Material, further inquiries can be directed to the corresponding author.
